# Desafíos en el manejo del sarcoma de Ewing en una paciente testigo de Jehová

**DOI:** 10.7705/biomedica.6720

**Published:** 2023-03-30

**Authors:** Carlos Julio Vargas-Potes, Diana Marcela Mendoza-Urbano, Luis Gabriel Parra-Lara, Ángela R. Zambrano

**Affiliations:** 1 Facultad de Ciencias de la Salud, Universidad Icesi, Cali, Colombia Universidad Icesi Universidad Icesi Cali Colombia; 2 Centro de Investigaciones Clínicas, Fundación Valle del Lili, Cali, Colombia Fundación Valle del Lili Cali Colombia; 3 Servicio de Hemato-Oncología, Departamento de Medicina Interna, Fundación Valle del Lili, Cali, Colombia Fundación Valle del Lili Cali Colombia

**Keywords:** sarcoma de Ewing, quimioterapia, anemia, transfusión de componentes sanguíneos, sarcoma, Ewing, drug therapy, anemia, blood component transfusion

## Abstract

El sarcoma de Ewing es una neoplasia de hueso y tejidos blandos, cuyo manejo se relaciona con toxicidad hematológica. Este aspecto representa un desafío médico y ético en los pacientes testigos de Jehová quienes, por sus creencias religiosas, rechazan la aplicación de hemoderivados, con riesgo de que se descontinúe la quimioterapia o de que se utilicen dosis subóptimas.

Se presenta el caso de una mujer colombiana de 34 años, testigo de Jehová, con diagnóstico de sarcoma de Ewing con estadificación clínica IIB (T_1_N_0_M_0_) en las regiones maxilar y mandibular izquierdas, tratada con quimioterapia, quien presentó un valor mínimo de hemoglobina de hasta 4,5 g/dl y tuvo indicación quirúrgica como parte del tratamiento. En estos pacientes, la decisión de practicar una transfusión comprende implicaciones éticas que requieren alternativas terapéuticas y un abordaje multidisciplinario.

## Abstract (en)

Ewing’s sarcoma is a bone and soft tissue neoplasm, whose management is related to hematological toxicity. This aspect represents a medical and ethical challenge in Jehovah’s Witnesses patients, who, due to their religious beliefs, reject the blood component transfusion, with the risk of discontinuing chemotherapy or using suboptimal doses.

We present the case of a 34-year-old Colombian woman, Jehovah’s Witness, diagnosed with Ewing’s sarcoma with clinical stage IIB (T Jehová han criticado esta prácticN_0_M_0_) in the left maxillary and mandibular regions, treated with chemotherapy, who presented a hemoglobin nadir of up to 4.5 g/dL, and surgical indication as part of the treatment. In these patients, the transfusion decision has ethical implications that require therapeutic alternatives and a multidisciplinary approach.

El sarcoma de Ewing abarca una familia de tumores de hueso y tejidos blandos, con translocación del gen *EWSR1*, con incidencia estimada de 2,93 casos por millón al año, principalmente en la segunda década de la vida, siendo raros después de los 30 años ^1^^-^^3^. El tratamiento estándar incluye quimioterapia intensiva, con riesgo de toxicidad hematológica ^4^.

Los testigos de Jehová son una comunidad religiosa de más de ocho millones de miembros, que manifiestan su rechazo a las transfusiones y quienes, al presentar una enfermedad oncológica, representan un desafío para el equipo tratante. La anemia se presenta hasta en el 34 % de los pacientes oncológicos que son testigos de Jehová ^5^, lo cual implica un riesgo de abandono o subdosificación de la quimioterapia, con compromiso de los resultados clínicos y la supervivencia.

No existe un consenso en el tratamiento estándar para el sarcoma de Ewing ni para el manejo alternativo con hemoderivados en los testigos de Jehová; además, la baja prevalencia de la enfermedad dificulta avanzar en las investigaciones clínicas. No está descrita la prevalencia de sarcoma de Ewing en los testigos de Jehová.

A continuación, se presenta el caso de una paciente testigo de Jehová con diagnóstico de sarcoma de Ewing, quien recibió tratamiento para su enfermedad, y que implicó un desafío ético y clínico para los profesionales de la salud.

## Presentación de caso

**Figura 1:**
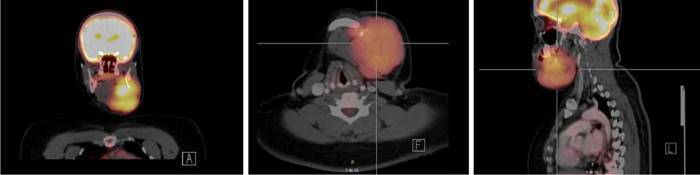
Tomografía por emisión de positrones que muestra una masa hipermetabólica en el maxilar inferior izquierdo, con compromiso óseo y componente de tejidos blandos (sarcoma de Ewing). Asimetría e hipermetabolismo en la amígdala palatina asociados con un ganglio IIA izquierdo.

Se trata de una paciente de 34 años, testigo de Jehová, sin comorbilidades, quien consultó por la aparición de una lesión dura e irregular en el maxilar izquierdo, con crecimiento progresivo, dolor y limitación para la apertura de la cavidad oral de aproximadamente 5 meses de evolución. En la tomografía por emisión de positrones (*Positron Emission Tomography, PET),* se encontró una masa hipermetabólica en el maxilar inferior izquierdo, con compromiso óseo y componente de tejidos blandos (figura 1), sin captaciones patológicas en las extremidades.

La biopsia de la lesión fue positiva para sarcoma de Ewing con estadificación clínica IIB (T_1_N_0_M_0_). Se inició tratamiento con ciclos de vincristina, doxorrubicina y ciclofosfamida, alternados con ifosfamida y etopósido (VAC/IE). En el primer ciclo, la paciente presentó caída de la hemoglobina a 9,5 g/dl; se aplicó pegfilgastrim al egreso. Después del segundo ciclo, presentó neutropenia febril de bajo riesgo y anemia grave (rechazó la indicación de transfusiones); se prescribió eritropoyetina, tres veces por semana a dosis de 2.000 UI/día. Completó siete ciclos de tratamiento, con hemoglobina mayor de 7 g/dl.

En el octavo ciclo, la paciente ingresó asintomática, pero con hemoglobina de 4,5 g/dl, sin neutropenia y con trombocitopenia leve, y función renal y electrolitos normales. Se indicó transfusión de hemoderivados, pero fueron rechazados nuevamente por la paciente, por lo cual se administró carboximaltosa de hierro, 1.000 mg intravenosos por dos días, más 30.000 UI subcutáneas de eritropoyetina. La nueva tomografía de cuello evidenció una lesión de menor tamaño respecto a los estudios previos. Se ofreció manejo quirúrgico, pero la paciente rechazó el procedimiento debido a la necesidad de transfusiones antes o durante la operación.

En la junta multidisciplinaria, se explicaron los riesgos y los beneficios asociados con el procedimiento. La paciente accedió por voluntad propia a la transfusión de hemoderivados, después de varios días de estancia hospitalaria. Al noveno día de hospitalización, con hemoglobina mayor de 8 g/dl, se le practicaron hemimandibulectomía izquierda y reconstrucción con colgajo microvascular. En el procedimiento se observó una reacción perióstica importante sin adenopatías, y no hubo complicaciones intraoperatorias ni anestésicas. Requirió atención en la unidad de cuidados intensivos durante su periodo posoperatorio, sin presentar nuevos eventos ni necesitar hemoderivados; se continuó su manejo en salas de hospitalización general y, finalmente, se le dio egreso tras 15 días de estancia hospitalaria, con un valor de hemoglobina de 6,8 g/dl.

### Consideraciones éticas

Durante todo el proceso se contó con la asesoría y el acompañamiento de la oficina jurídica y el comité de ética hospitalaria.

Se cumplió con el proceso de consentimiento informado con la paciente.

## Discusión

La religión ofrece perspectivas poderosas sobre el sufrimiento, la pérdida y la muerte, y muchas personas están profundamente comprometidas con sus tradiciones religiosas. El valor de la religión toma un papel protagónico en contextos de enfermedad y muerte. Si bien, los profesionales de la salud no son expertos en doctrina religiosa, se enfrentan a situaciones que les obligan a discutir preocupaciones religiosas con sus pacientes, lo cual no debe ser ignorado ^6^^,^^7^. En este caso de la paciente testigo de Jehová, la discusión fue sobre la transfusión de hemocomponentes para la intervención quirúrgica.

La corporación religiosa *Watchtower Society* introdujo la política de rechazo de la transfusión de sangre en 1945, incluso en casos de hemorragia potencialmente mortal. Desde 1961, la iglesia lo ha hecho cumplir expulsando a los miembros que no se han arrepentido y que deliberadamente aceptan componentes sanguíneos prohibidos. Luego, la iglesia instruye a los otros miembros para que excluyan y eviten al individuo expulsado. Ellos creen que, para Dios, la sangre representa la vida y, por consiguiente, se abstienen de la sangre (trasfusiones) por respeto a Dios, quien dio la vida.

“[...] Porque la vida de toda carne es su sangre; por tanto, he dicho a los hijos de Israel: No comeréis la sangre de ninguna carne, porque la vida de toda carne es su sangre; cualquiera que la comiere será cortado [...]”. Levítico 17:14

Otras menciones bíblicas incluyen:

“[...] Pero carne con su vida, que es su sangre, no comeréis [...]”. Génesis 9:4

“[...] Y cualquier hombre de la casa de Israel, o de los extranjeros que peregrinan entre ellos, que coma sangre alguna, yo pondré mi rostro contra esa persona que coma sangre y la talaré de entre su pueblo [...]”. Levítico 17:10

“[...] Solamente que te mantengas firme en no comer sangre; porque la sangre es la vida, y no comerás la vida juntamente con su carne [...]”. Deuteronomio 12:23

“[...] Porque ha parecido bien al Espíritu Santo, y a nosotros, no imponeros ninguna carga más que estas cosas necesarias: que os abstengáis de lo sacrificado a ídolos, y de sangre, y de lo estrangulado y de fornicación; de tales cosas si os guardáis, bien haréis. Pasadlo bien [...]”. Hechos 15:28-29

Por consiguiente, la transfusión de hemoderivados implica una violación absoluta de las leyes de Dios y la imposibilidad de trascender al Paraíso después de la muerte, además de una potencial sanción social por parte de su comunidad ^8^^,^^9^. Sin embargo, disidentes internos de los testigos de Jehová han criticado esta práctica por coaccionar a quienes tienen puntos de vista divergentes sobre el tema y compromete la toma de decisiones autónomas en la atención médica. Como resultado, los profesionales de la salud deben conocer las opciones terapéuticas alternativas para los testigos de Jehová y tratar a estos pacientes de forma individualizada ^10^.

En estas circunstancias, el médico suele enfrentarse a una encrucijada entre la obligación de preservar la vida o acatar la autonomía del paciente ^11^. Lo anterior lleva a dos situaciones: por un lado, la falta de acción puede dar lugar a cargos penales de negligencia o, incluso, en caso de muerte del paciente, de homicidio culposo; por otro lado, podría dar lugar a denuncias penales de atentado contra la integridad física o a reclamaciones de daños y perjuicios por parte del paciente, por vulneración del derecho a la libre determinación ^12^.

Cuando el paciente se encuentra en una situación *in extremis*, existe una importante controversia por el paternalismo médico que se puede presentar en pro del principio de beneficencia. El paternalismo médico se debería aceptar como un beneficio para el paciente solo en ciertas situaciones (riesgo significativo, daño prevenible), en las que se considera que los beneficios superarán los riesgos para el paciente ^13^. En otros casos, aquellos de menor complejidad, se puede propender a influir sobre las decisiones del paciente y tener impactos psicosociales negativos ^14^.

A veces, el médico puede optar por derivar al paciente o su familia a un colega comprensivo o a un religioso, o por establecer un diálogo para conocer sus creencias y discutir si estas pueden afectar su atención. Dicho diálogo debe estar marcado por la sabiduría, la franqueza, el respeto y la información correcta. Se puede buscar información de la iglesia o de otras fuentes ^6^.

El sarcoma de Ewing es una neoplasia de hueso y tejidos blandos poco frecuente, especialmente después de los 30 años ^3^, que se localiza en cabeza o cuello solo en 1 a 9 % de los casos ^15^. Esta paciente testigo de Jehová presentó este diagnóstico de baja prevalencia, con particularidades que inciden en los resultados, incluyendo el rechazo de las transfusiones que limita las opciones de manejo. En los testigos de Jehová con diagnóstico de neoplasias de órgano sólido hay tasas de supervivencia a 5 años de hasta el 70 %, sin que la quimioterapia o la cirugía se asocien con complicaciones graves, estando la mortalidad más relacionada con la progresión de la enfermedad. Sin embargo, en las cohortes retrospectivas no hay un número significativo de pacientes testigos de Jehová con tumores de hueso o tejidos blandos ^16^.

Un estudio retrospectivo de cohorte, realizado en Australia, reportó una incidencia de anemia del 34 % entre los pacientes testigos de Jehová con neoplasia hematológica o de órgano sólido. Todos los pacientes rechazaron las transfusiones cuando les fueron ofrecidas, incluso aquellos con anemia sintomática o amenazante para la vida, llegando a presentarse hasta un 24 % de discontinuación a la quimioterapia. Sin embargo, no se encontró que la mortalidad fuera directamente atribuible a la anemia o al rechazo de los hemoderivados, sino probablemente, a la discontinuación de la quimioterapia y la administración de agentes estimulantes de eritropoyesis ^5^. La transfusión de hemoderivados en la paciente se llevó a cabo antes de la cirugía, por anemia grave, y después de la intervención quirúrgica y de la quimioterapia.

Para el sarcoma de Ewing localizado se han descrito mayores tasas de supervivencia libre de eventos a 5 , en los pacientes en esquema intensivo de quimioterapia VAC/IE (cada 2 semanas) *Vs.* esquema estándar (3 semanas), sin aumento de la toxicidad o necesidad de reducir las dosis ^4^^,^^17^. En este caso, se inició un esquema de VAC/IE en los primeros 30 días después de la confirmación histológica, y se completaron 7 ciclos continuos del esquema terapéutico estándar, sin que se presentaran efectos adversos que contraindicaran su uso. Sin embargo, la toxicidad hematológica es un aspecto relevante en el manejo de los pacientes testigos de Jehová, pues se ha visto una toxicidad hematológica de grado 3/4 en el 16 % de los ciclos de VAC/IE ^18^.

A la fecha, no existen terapias superiores al conjunto de estos agentes farmacológicos que se utilizan desde hace varias décadas. Para el momento en que la paciente se sometió a la cirugía, llevaba un retraso de tratamiento mayor de cuatro semanas, con riesgo de disminución en la supervivencia. Además, hasta el 25 % de los pacientes con enfermedad localizada recaen después de completar el esquema y el 70 % presentan la recaída en los dos primeros años después del diagnóstico ^19^.

La incidencia de transfusiones sanguíneas perioperatorias en cirugía de cabeza y cuello, es heterogénea (12-84 %) ^20^. En cirugías reconstructivas con colgajos libres, es particularmente importante la oxigenación de los injertos para su viabilidad, por lo que se han propuesto puntos de corte para la necesidad de transfusión en estos procedimientos, con hemoglobina menor de 7,0 g/dl o hematocrito menor del 25 %, sin haber consenso en este aspecto ^21^. El tipo de colgajo no tiene impacto estadísticamente significativo para la tasa de transfusiones sanguíneas ^22^. En el caso de esta paciente, se planteó una meta de hemoglobina de 8,0 g/dl en el periodo prequirúrgico, sin necesidad de administrar hemoderivados en el periodo posquirúrgico ni durante el resto de estancia en la unidad de cuidados intensivos o la sala de hospitalización.

Los consensos entre los gremios médicos y representantes de los pacientes testigos de Jehová han organizado estrategias para una “medicina y cirugía sin sangre”; incluyen el uso de fármacos para mejorar la capacidad de incrementar los niveles de hemoglobina, evitar pérdidas intraoperatorias de sangre, reducir al máximo el número de flebotomías y minimizar el consumo de oxígeno. Se ha evidenciado que concentraciones de hemoglobina de 4,0 a 4,5 g/dl (las cuales llegó a presentar la paciente, sin síntomas ni compromiso hemodinámico) constituyen un rango en que se requieren medidas protectoras agresivas ^23^.

Es importante tener en cuenta que los testigos de Jehová rechazan la transfusión de glóbulos rojos, leucocitos, plaquetas y plasma, pero su aceptación de “fracciones” sanguíneas (crioprecipitado, albúmina y factores de coagulación) es variable y queda a discreción de cada persona. Otras estrategias en este enfoque incluyen el uso de eritropoyetina, hierro parenteral, ácido fólico, vitamina B_12_, oxígeno, reposo estricto en cama, soporte nutricional, tromboprofilaxis y profilaxis gástrica ^24^. En este caso, se aplicaron varias de estas estrategias; en el manejo perioperatorio la necesidad de emplear recuperación de células (*cell saver),* hemodilución hipervolémica e hipotermia fue sustituida por la aceptación de la paciente de las transfusiones preoperatorias e intraoperatorias.

El hecho de que haya riesgo de muerte por el rechazo de una práctica que es común, aunque no inocua, es el verdadero desafío ^25^. Si bien los profesionales de la salud buscan beneficiar los pacientes, cada caso que involucre testigos de Jehová se debe individualizar, respetando sus creencias y su capacidad de decisión. Particularmente en los testigos de Jehová, hay diversidad de creencias, por lo que es importante conocerlas antes del proceso terapéutico y durante el mismo, educar al paciente, prever potenciales efectos adversos y complicaciones, y tener listas alternativas en caso de desistimientos definitivos del paciente; además, conviene apoyarse en un comité de ética y el servicio jurídico para incluirlos en el plan de manejo multidisciplinario.

## Conclusión

La toma de decisiones con respecto a la necesidad de transfundir productos sanguíneos es un aspecto que hay que valorar de manera individualizada en las fases de diagnóstico y tratamiento de la enfermedad oncológica en el paciente testigo de Jehová. El cumplimiento de los principios éticos, incluyendo el respeto a la libertad de creencias religiosas, debe llevar a buscar alternativas para mitigar y controlar eventos adversos de los tratamientos que se aplican con el objetivo de beneficiar al paciente.
